# Safety and efficacy of echinocandin antifungal agents in *Candida albicans* endophthalmitis

**DOI:** 10.1128/aac.01670-25

**Published:** 2026-03-23

**Authors:** Yue Zhang, Yi Tang, Yanjie Zhou, Hong Wu

**Affiliations:** 1 Department of Ophthalmology, the Second Hospital of Jilin University 626989 , Changchun, Jilin, China; 2 Department of Ophthalmology, Changzhi People’s Hospital, Changzhi, Shanxi, China; University of Iowa, Iowa City, Iowa, USA

**Keywords:** fungal endophthalmitis, *Candida albicans*, rezafungin, anidulafungin, liposomal amphotericin B, voriconazole

## Abstract

To evaluate the safety and efficacy of echinocandins (rezafungin, RZF; anidulafungin, AFG) in *Candida albicans (C. albicans*) endophthalmitis, with a particular focus on the novel drug RZF. In vitro safety was assessed by CCK-8 and live/dead staining on ARPE-19, Müller, and RAW 264.7. For *in vivo* safety, we administered therapeutic doses via intravitreal injection in rabbit eyes and performed fundus photography and histopathological analysis on day 3. Antifungal effect evaluation included minimum inhibitory concentration (MIC) determination, biofilm inhibition, and structural damage observation (confocal laser scanning microscopy, CLSM; scanning/transmission electron microscopy, SEM/TEM). We measured inflammatory responses via TNF-α/IL-1β qRT-PCR in RAW 264.7 cells. A rabbit *C. albicans* endophthalmitis model was established. At 48 h post-infection, rabbits received intravitreal injections of liposomal amphotericin B (L-AmB, 10 μg), voriconazole (VCZ, 50 μg), RZF (35 μg), AFG (35 μg), or saline (control). Clinical scores, ocular fungal burden, aqueous humor TNF-α, and histopathological scores were assessed. Echinocandins showed lower IC50 values *in vitro*, yet maintained acceptable safety at clinically relevant low concentrations compared with traditional drugs, with no significant tissue toxicity *in vivo*. RZF and AFG exhibited lower MICs (0.016–0.03125 μg/mL) than L-AmB (4 μg/mL) and VCZ (0.125–0.25 μg/mL). All four drugs inhibited biofilm formation, disrupted fungal structures, and downregulated inflammatory responses. *In vivo*, RZF/AFG reduced clinical and histopathological scores, fungal burden, and TNF-α levels, performing similarly to traditional drugs. RZF and AFG demonstrate potent antifungal activity against *C. albicans* comparable to conventional drugs, along with significant anti-inflammatory effects and acceptable safety profiles.

## INTRODUCTION

*Candida albicans (C. albicans*) endophthalmitis is a severe intraocular infection that threatens vision. Exogenous fungal endophthalmitis may occur after ocular surgery, corneal trauma, or dissemination from adjacent tissues (1). Endogenous *C. albicans* infection typically arises when immunity is compromised, allowing *C. albicans* to invade the bloodstream and spread to the eye and is often associated with risk factors such as immunosuppression, prolonged catheterization, corticosteroid use, and abdominal surgery (2). Clinically, *Candida* endophthalmitis is characterized by choroidoretinitis and vitritis. Without timely intervention, it can progress to vitreous abscess, retinal necrosis, or even tractional retinal detachment, leading to irreversible vision loss.

Currently, traditional antifungal drugs have significant limitations. Liposomal amphotericin B (L-AmB) remains the cornerstone of treatment due to its broad-spectrum antifungal activity, but its severe systemic nephrotoxicity significantly limits clinical use, especially given the risk of retinal toxicity with intravitreal administration (3). Voriconazole (VCZ) has good intraocular penetration, but issues such as rising resistance among *Candida* species, a short intraocular half-life, and drug interactions constrain its efficacy (4–6). Notably, mature *C. albicans* biofilms—a key virulence factor for persistent infection and treatment resistance—have not been reliably overcome by major traditional antifungals (e.g., polyenes such as amphotericin B and its lipid formulations, or azoles like VCZ) (7). Therefore, there is an urgent need to develop novel antifungal agents that possess safety, potent anti-biofilm activity, and efficacy against resistant strains.

Echinocandin antifungals act by inhibiting fungal cell wall β-(1, 3)-D-glucan synthesis, thereby exerting potent activity against *Candida* species (including azole-resistant strains) with high safety (8). Currently available echinocandins include caspofungin, micafungin, anidulafungin (AFG), and the new-generation rezafungin (RZF). Karagoz *et al*. demonstrated in a study that AFG is effective in a *Candida* endophthalmitis model (9). As a new-generation echinocandin, RZF has an extended half-life and greater stability (10). Preclinical studies suggest that it has anti-biofilm potential (11, 12), and systemic administration has shown good efficacy and safety (13). In March 2023, it was approved by the US FDA for the treatment of candidemia and invasive candidiasis (14). However, systemic administration may not achieve an effective therapeutic concentration in the eye. Moreover, the local safety, anti-inflammatory properties, and efficacy of intravitreal RZF in treating *C. albicans* endophthalmitis have yet to be systematically evaluated.

This study aimed to address this knowledge gap by comprehensively evaluating the *in vitro* and *in vivo* antifungal activity and safety of RZF and AFG (with particular focus on RZF), and comparing their efficacy with traditional drugs (L-AmB and VCZ).

## RESULTS

### Echinocandins exhibit good cytocompatibility

**Fig 1:**
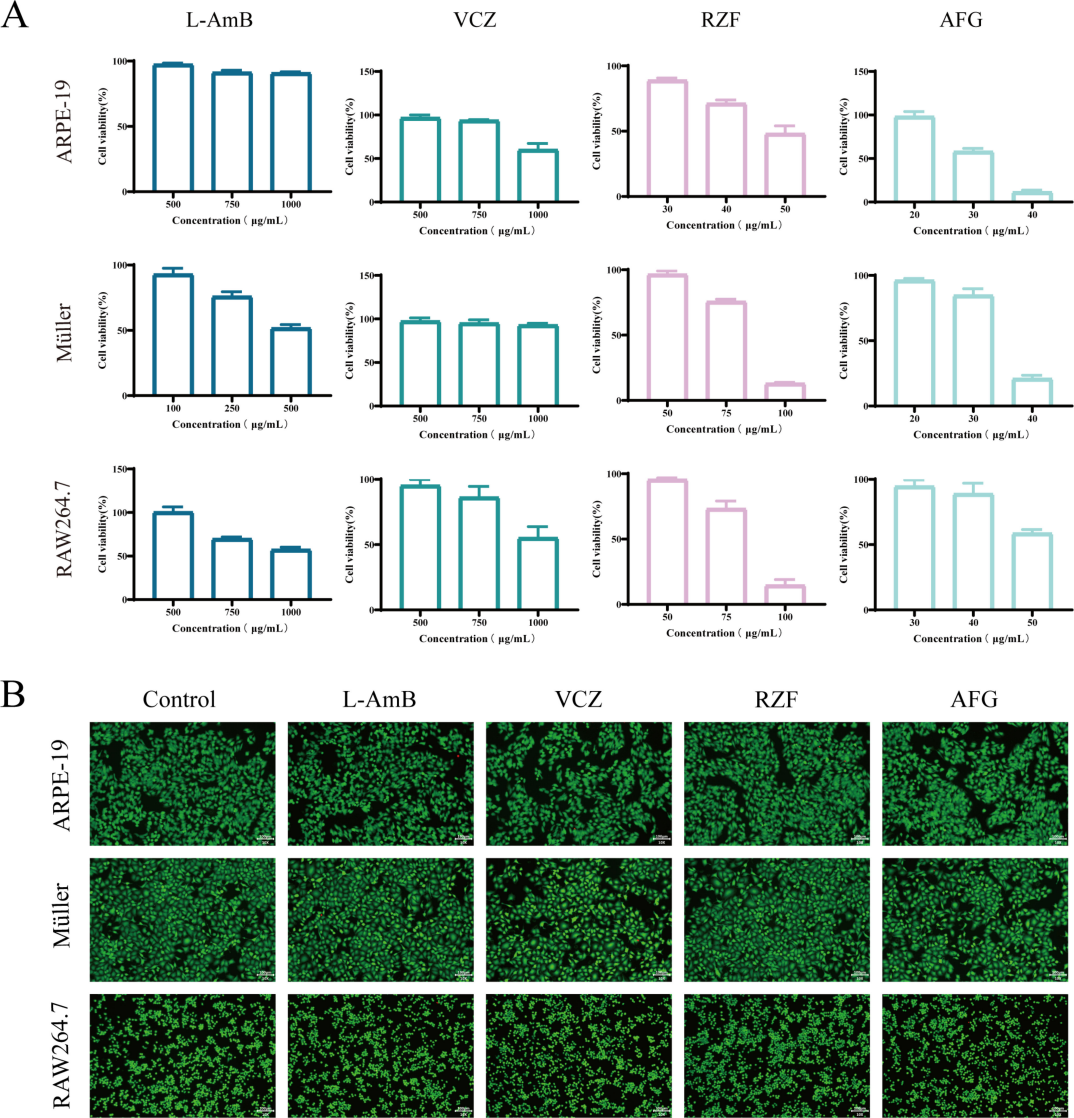
(**A**) Cell viability of ARPE-19, Müller, and RAW 264.7 cells treated with four drugs at different concentrations as determined by the CCK-8 assay. (**B**) Live/dead staining of ARPE-19, Müller, and RAW 264.7 cells with Calcein-AM/PI after 3 days of co-culture with the highest drug concentration that maintains 90% cell viability (scale bar: 100 μm). Concentrations used in ARPE-19: L-AmB: 1,000 μg/mL; VCZ: 750 μg/mL; RZF: 30 μg/mL; and AFG: 20 μg/mL. Concentrations used in Müller: L-AmB: 100 μg/mL; VCZ: 1,000 μg/mL; RZF: 50 μg/mL; and AFG: 20 μg/mL. Concentrations used in RAW 264.7: L-AmB: 500 μg/mL; VCZ: 500 μg/mL; RZF: 50 μg/mL; and AFG: 30 μg/mL.

The CCK-8 results showed that in ARPE-19 cells, the half-maximal inhibitory concentration (IC50) of L-AmB and VCZ exceeded 1,000 μg/mL, compared to 40–50 μg/mL for RZF and 30–40 μg/mL for AFG. In Müller cells, IC50 was approximately 500 μg/mL for L-AmB, over 1,000 μg/mL for VCZ, and 75–100 μg/mL and 30–40 μg/mL for RZF and AFG, respectively. In RAW 264.7 cells, the IC50 of L-AmB and VCZ was over 1,000 μg/mL, while the IC50 of RZF and AFG was 75–100 μg/mL and approximately 50 μg/mL, respectively (Fig. 1A). Notably, the higher IC50 values observed for L-AmB are consistent with its liposomal formulation, which is known to reduce nonspecific interactions with mammalian cholesterol-containing membranes and thereby lower host-cell toxicity. In addition, L-AmB concentrations are reported as the nominal total amphotericin B content in the formulation, which does not necessarily reflect the free drug fraction available to cells *in vitro* (15). Despite lower IC50 values for RZF/AFG than for L-AmB/VCZ, the IC50-to-MIC ratios for RZF/AFG were generally around or above 10³, indicating a wide *in vitro* selectivity margin relative to antifungal potency. Fluorescence microscopy confirmed that after 3 days of culture at the highest drug concentration that maintained 90% cell viability, the morphology and fluorescence intensity of living cells were normal, and no obvious dead cells were observed (Fig. 1B). The live-dead staining results were consistent with the CCK-8 results, indicating that all four drugs exhibited minimal cytotoxicity at the tested concentrations.

### Echinocandins exhibit favorable *in vivo* biocompatibility

**Fig 2:**
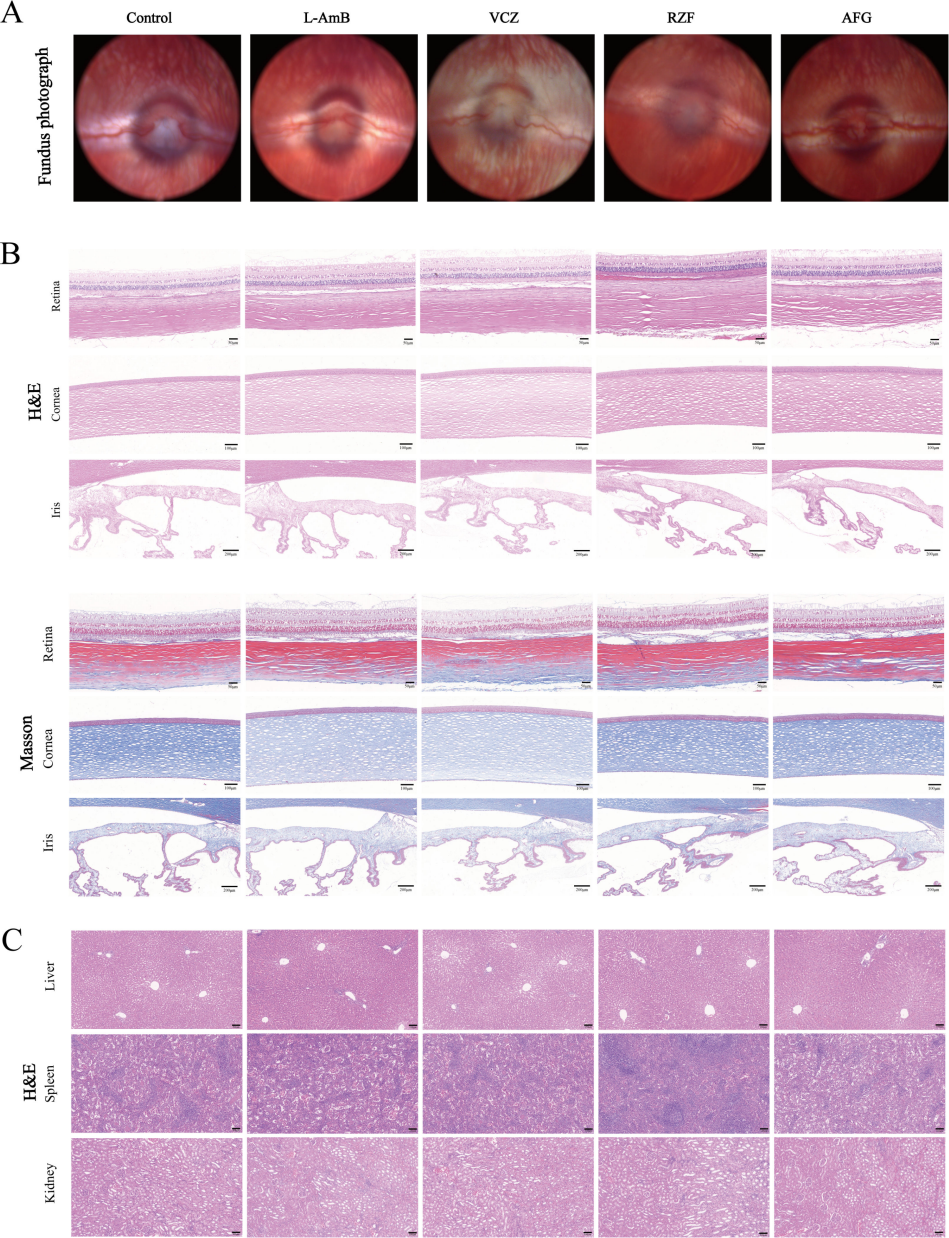
(**A**) Fundus color photographs of rabbit eyes at 3 days after a single intravitreal injection of 0.1 mL saline, 10 μg/0.1 mL L-AmB, 50 μg/0.1 mL VCZ, 35 μg/0.1 mL RZF, and 35 μg/0.1 mL AFG, respectively. (**B**) H&E and Masson staining results of the retina, cornea, and iris of rabbit eyes at 3 days after a single intravitreal injection of the four drugs and saline, respectively (scale bars: retina 50 μm, cornea 100 μm, iris 200 μm). (**C**) H&E staining results of the liver, spleen, and kidney at 3 days after a single intravitreal injection of the four drugs and saline, respectively (scale bar: 100 μm) (*n* = 1/ group).

Compared with healthy controls, no detectable abnormalities were observed in fundus photography, ocular tissues, and major peripheral organs in any drug-treated group. Fundus photography revealed that none of the four drugs induced significant intraocular toxic responses (Fig. 2A). Histopathology (H&E and Masson-stained) of ocular tissues (retina, cornea, and conjunctiva) revealed intact retinal layers, organized corneal collagen, normal iris epithelium, and absence of inflammation or fibrosis (Fig. 2B). Histological evaluations of the liver, spleen, and kidneys demonstrated clear hepatic lobular architecture, distinct demarcation between the red and white pulp of the spleen, and absence of vacuolar degeneration in the glomerular basement membrane and renal tubular epithelial cells (Fig. 2C). These findings indicate that at the established therapeutic concentrations, RZF and AFG, along with the conventional agents L-AmB and VCZ, all displayed excellent ocular and systemic safety profiles in the rabbit model.

### Echinocandins have low minimum inhibitory concentrations

The OD_600_ of each well was measured using a microplate reader, and the results were recorded (Table 1).

**TABLE 1:** MICs of *C. albicans* (ATCC 10,231)

Drugs	MICs (μg/mL)
L-AmB	4
VCZ	0.125–0.25
RZF	0.016–0.03125
AFG	0.016–0.03125

### Echinocandins can effectively inhibit the formation of *C. albicans* biofilms

**Fig 3:**
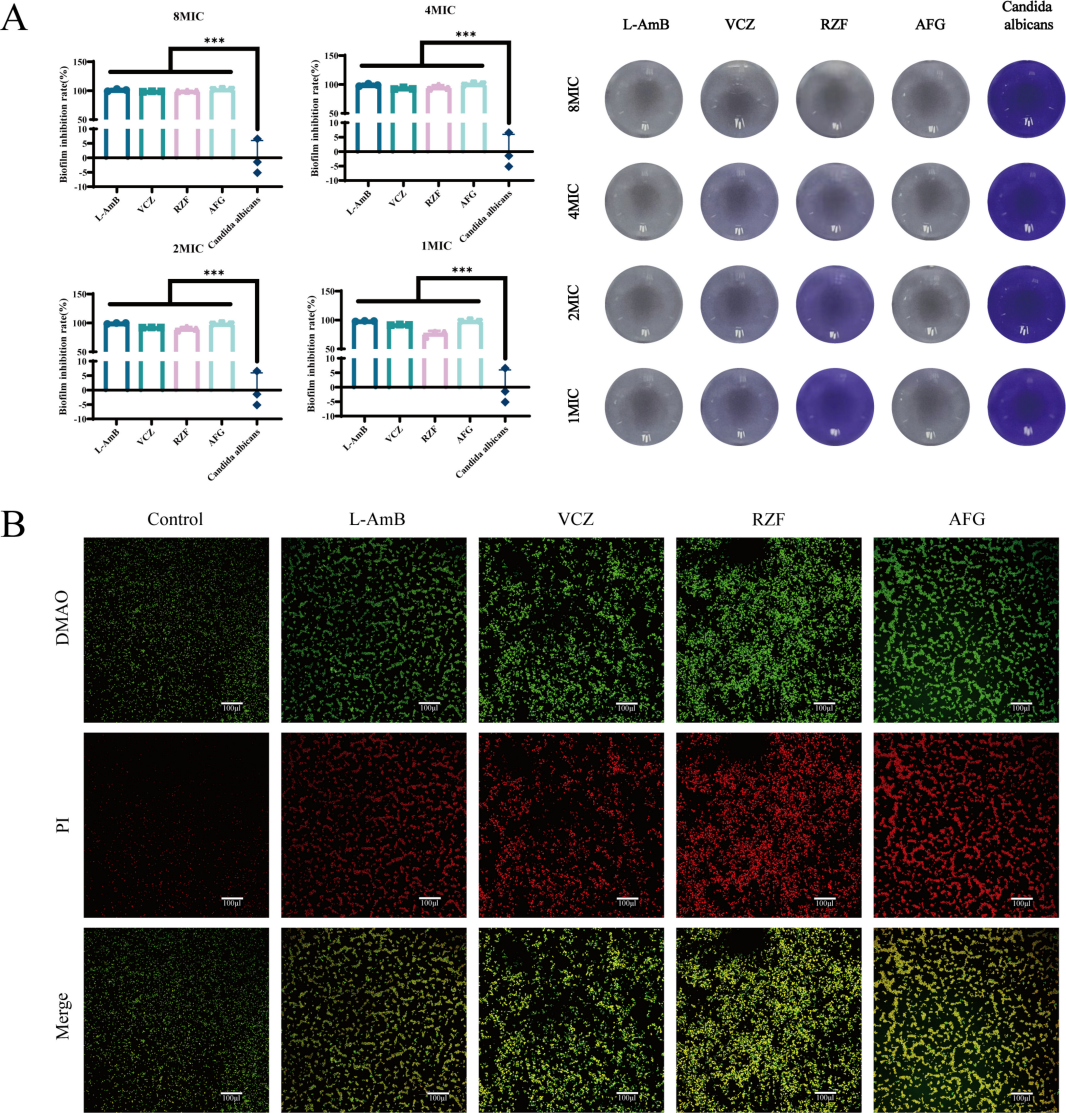
(**A**) Inhibition rates of L-AmB, VCZ, RZF, and AFG on *C. albicans* biofilm formation at different concentrations as determined by crystal violet staining. (**B**) Confocal microscopic images of *C. albicans* biofilms developed in the presence of the four drugs at 4× MIC and co-stained with DMAO and PI (scale bar: 100 μm). *** P < 0.001.

All four drugs (L-AmB, VCZ, RZF, and AFG) at 1×, 2×, 4×, or 8× MICs significantly inhibited the formation of *C. albicans* biofilms, and the inhibitory effect was concentration-dependent (Fig. 3A). At 8× MIC, the inhibition rates of L-AmB, VCZ, RZF, and AFG on biofilm formation were 100%, 98.64%, 99.07%, and 100%, respectively; at 4× MIC, the inhibition rates were 99.01%, 93.12%, 94.98%, and 100%, respectively; at 2× MIC, the inhibition rates were 99.26%, 92.13%, 89.65%, and 98.26%, respectively; at 1× MIC, the inhibition rates were 98.14%, 91.69%, 76.26%, and 98.02%, respectively. These results confirm that both echinocandins (RZF, AFG) and traditional antifungals (L-AmB, VCZ) can significantly inhibit the formation of *C. albicans* biofilms at different concentrations.

The membrane integrity of *C. albicans* treated with the four drugs was further evaluated using DMAO and PI staining, followed by CLSM (Fig. 3B). In the control group, only minimal PI staining was observed. In contrast, treatment with 4× MIC of any drug significantly increased PI fluorescence, indicating substantial membrane damage.

### Electron microscopy reveals qualitative ultrastructural injury in *C. albicans* after antifungal exposure

**Fig 4:**
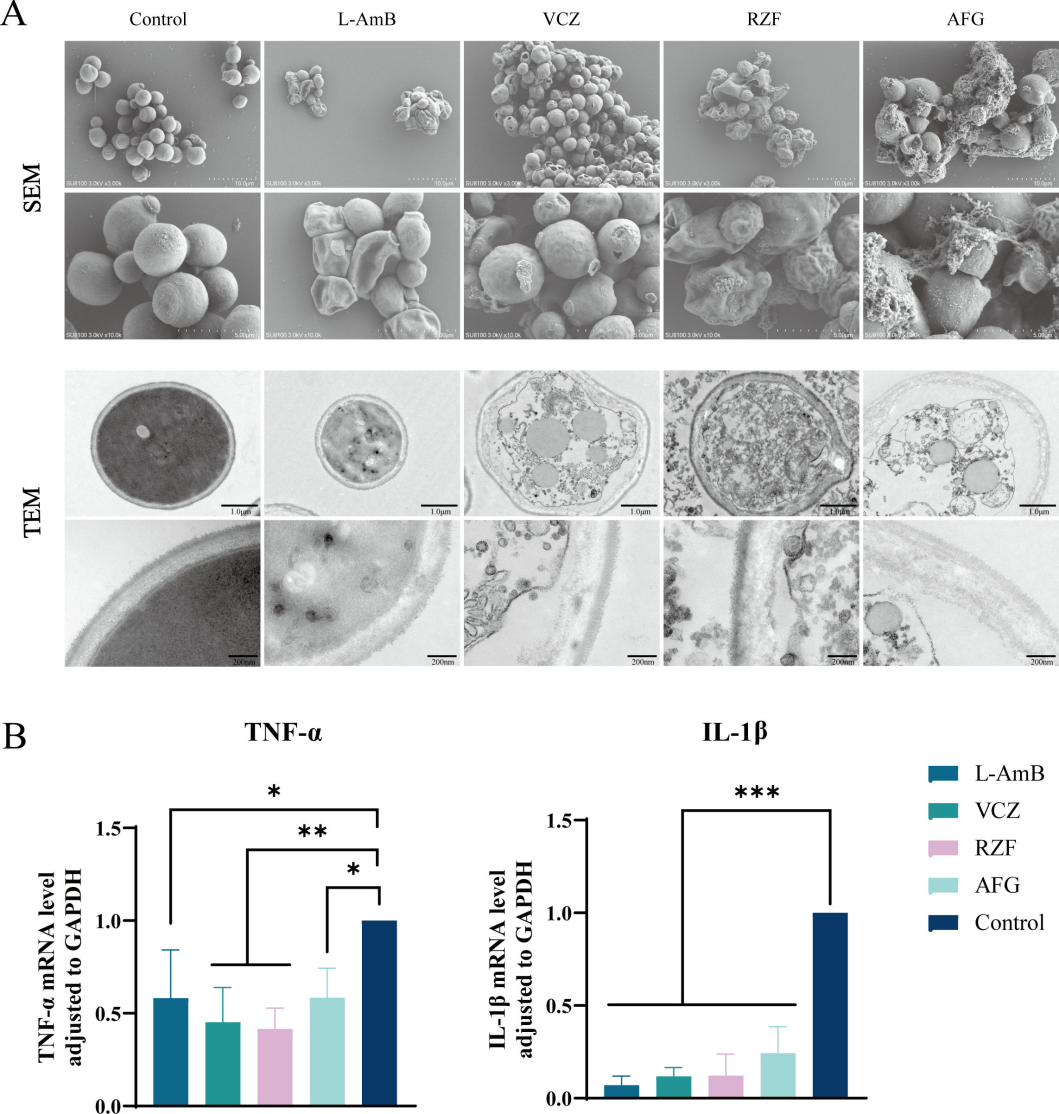
(**A**) SEM and TEM images of *C. albicans* after 48 h exposure to L-AmB (16 μg/mL), VCZ (1.0 μg/mL), RZF (0.125 μg/mL), or AFG (0.125 μg/mL) (4×MIC) (SEM scale bars: 10 μm/5 μm; TEM scale bars: 1 μm/200 nm). (**B**) mRNA expression levels of TNF-α and IL-1β after co-culture of *C. albicans* and RAW 264.7 cells treated with L-AmB (160 μg/mL), VCZ (10 μg/mL), RZF (1.25 μg/mL), or AFG (1.25 μg/mL) for 2 h (40× MIC). * *P* < 0.05, ** *P* < 0.01, *** *P* < 0.001.

SEM analysis demonstrated that untreated *C. albicans* cells exhibited a smooth and intact surface morphology. In contrast, cells exposed to L-AmB, VCZ, RZF, or AFG showed irregular contours accompanied by surface roughness and collapse. TEM observations further revealed that after 48 h of treatment, the cell envelopes were damaged with concomitant disorganization of intracellular structures (Fig. 4A). These ultrastructural findings provide qualitative evidence for the cellular injury induced by the antifungal agents.

### Antifungal co-treatment reduces macrophage pro-inflammatory cytokine transcription during short-term *C. albicans* stimulation

qRT-PCR analysis showed that after RAW 264.7 cells were co-cultured with *C. albicans* and treated with L-AmB (160 μg/mL), VCZ (10 μg/mL), RZF (1.25 μg/mL), or AFG (1.25 μg/mL) for 2 h, the mRNA levels of TNF-α and IL-1β were significantly reduced compared to the untreated control (Fig. 4B). Because drugs were present during stimulation, these data represent a net reduction in cytokine induction under co-treatment conditions, which may reflect reduced fungal stimulatory capacity/viable burden and/or direct immunomodulatory effects of the antifungals.

### Rabbit model of *C. albicans* endophthalmitis

**Fig 5:**
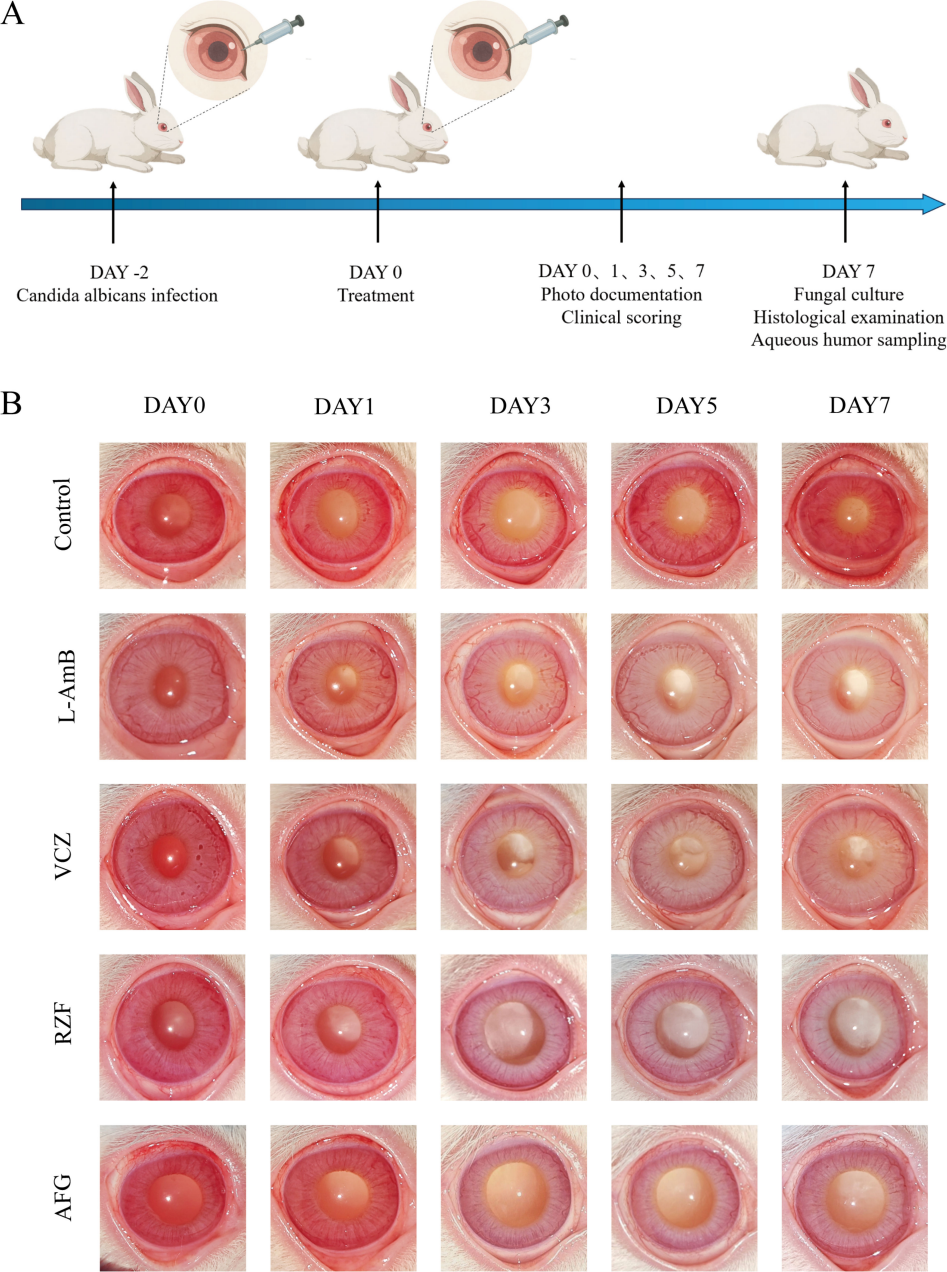
(**A**) Timeline for modeling, therapeutic intervention, and evaluation of *C. albicans* endophthalmitis infection. (**B**) Representative external photographs of infected rabbit eyes treated with intravitreal normal saline, L-AmB, VCZ, RZF, or AFG at Day 0 (baseline, immediately prior to dosing at 48 h post-infection), Day 1, Day 3, Day 5, and Day 7 (*n* = 5/ group).

The experimental timeline is shown in Fig. 5A. Day 0 images (Fig. 5B) were acquired immediately prior to intravitreal dosing at 48 h post-infection to document baseline disease severity. A representative external photograph of a healthy, uninfected rabbit eye imaged under the same conditions is provided in Fig. S1 as a normal reference. At 48 h post-infection (Day 0), all eyes exhibited typical endophthalmitis, including corneal edema, mild conjunctival hyperemia and edema, pronounced iris hyperemia, and mild vitreous opacity. Control eyes worsened over time, showing diffuse corneal edema, conjunctival exudation, iris synechia, and complete vitreous opacity with loss of the red reflex by Day 7. All four drug treatments effectively halted disease progression. L-AmB performed better than the other three groups in corneal repair speed and iris inflammation control. Mild iris hyperemia persisted in the AFG group on Day 7, suggesting a potentially delayed anti-inflammatory effect. However, all treatment groups eventually showed comparable outcomes by the end of the study (Fig. 5B).

**Fig 6:**
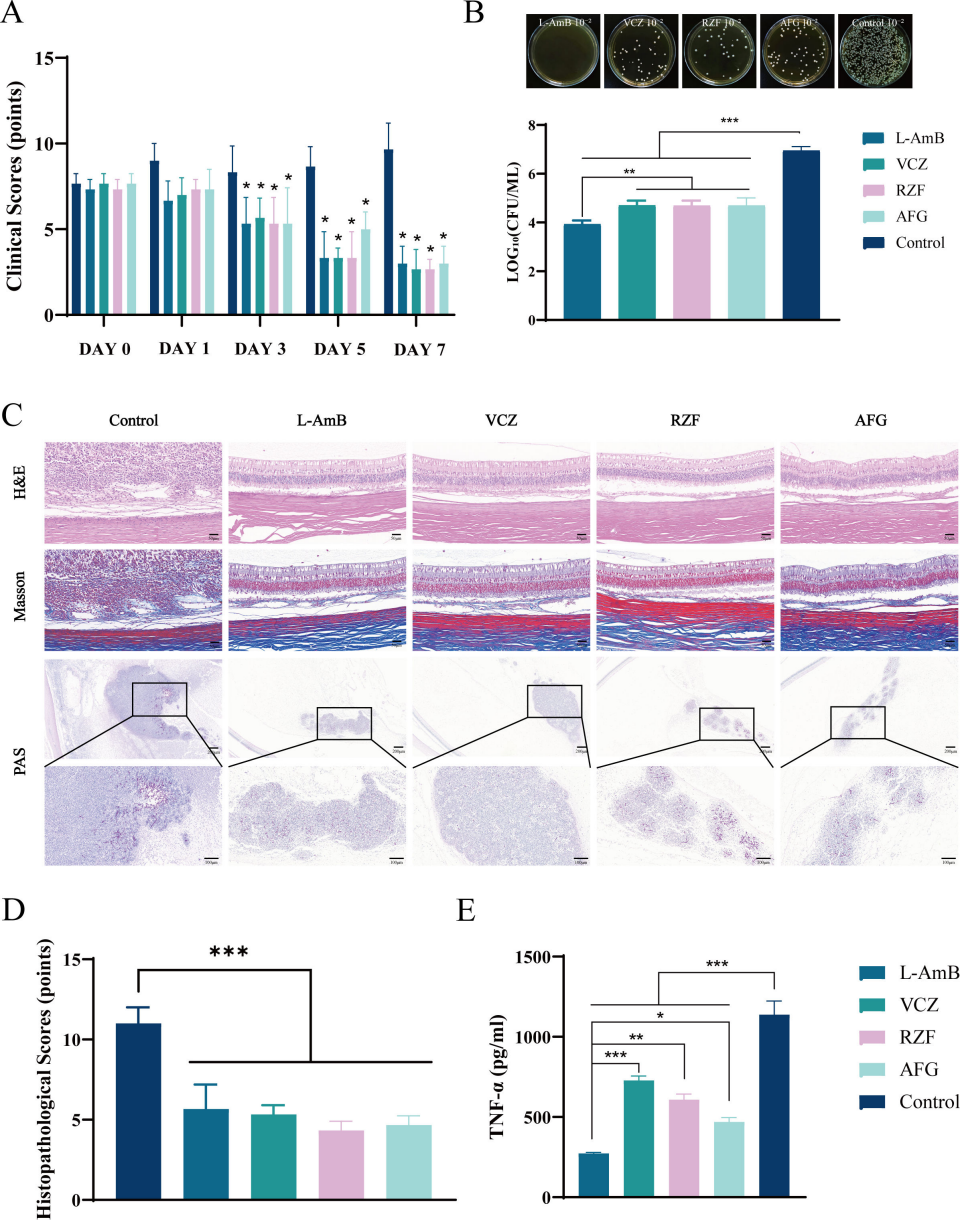
(**A**) Clinical scoring results of each group at Day 0, Day 1, Day 3, Day 5, and Day 7 after drug injection (*n* = 5/ group). (**B**) Results of dilution plating counting (CFU) of eyes allocated for fungal burden analysis at Day 7 (*n* =3 eyes/group). (**C**) Representative retinal H&E (scale bar: 50 μm), Masson (scale bar: 50 μm), and PAS staining (scale bars: 200 μm/100 μm) of eyes allocated for histology at Day 7 (*n* =2 eyes/group). (**D**) Pathological section scoring results at Day 7 (*n* =2 eyes/group). (**E**) TNF-α expression levels in aqueous humor at Day 7 as determined by ELISA (*n* = 5/ group). * *P* < 0.05, ** *P* < 0.01, *** *P* < 0.001.

Clinical scores indicated no significant differences among any groups on Days 0 and 1 (*P* > 0.05). On days 3, 5, and 7, no significant differences were observed between the RZF/AFG groups and the traditional drug groups (L-AmB/ VCZ) (*P* > 0.05), whereas all four treatment groups differed significantly from the control group (*P* < 0.05) (Table 2; Fig. 6A).

**TABLE 2:** Clinical scoring results of endophthalmitis in each group

Groups	Time points				
	Day 0	Day 1	Day 3	Day 5	Day 7
Control	7.67 ± 0.58	9.00 ± 1.00	8.33 ± 1.53	8.67 ± 1.15	9.67 ± 1.53
L-AmB	7.33 ± 0.58	6.67 ± 1.15	5.33 ± 1.53	3.33 ± 1.53	3.00 ± 1.00
VCZ	7.67 ± 0.58	7.00 ± 1.00	5.67 ± 1.15	3.33 ± 0.58	2.67 ± 1.15
RZF	7.33 ± 0.58	7.33 ± 0.58	5.33 ± 1.53	3.33 ± 1.53	2.67 ± 0.58
AFG	7.67 ± 0.58	7.33 ± 1.15	5.33 ± 2.08	5.00 ± 1.00	3.00 ± 1.00

Fungal burden analysis showed significantly reduced colony counts in all treatment groups compared to the control (Fig. 6B). Notably, the L-AmB group exhibited significantly lower residual CFU than the VCZ, RZF, and AFG groups. All four treatment groups achieved fungicidal rates exceeding 99% (Table 3).

**TABLE 3:** Results of dilution plating counting and fungicidal rates in different groups

Groups	LOG10 (CFU/mL)	Fungicidal rates (%)
Control	6.95 ± 0.17	0
L-AmB	3.93 ± 0.15	99.905 ± 0.035
VCZ	4.70 ± 0.20	99.428 ± 0.232
RZF	4.69 ± 0.20	99.436 ± 0.251
AFG	4.69 ± 0.32	99.389 ± 0.333

Pathological analysis revealed that in the control group, diffuse inflammatory cell infiltration, fibrinous exudation, and necrotic debris were observed in the vitreous cavity, and the retina presented full-thickness necrosis. In contrast, all treatment groups (L-AmB, VCZ, RZF, and AFG) showed focal inflammatory cells scattered in the vitreous cavity, with the retinal tissue structure roughly intact and no necrosis. PAS staining revealed that a large number of dense purple-red *C. albicans* clumps were present in the vitreous cavity of the control group, while all drug groups exhibited markedly reduced fungal loads. PAS staining revealed abundant dense *C. albicans* aggregates in the vitreous cavity of the control group, whereas all drug-treated groups showed markedly reduced fungal loads. In these qualitative sections, hyphal elements appeared less extensive in treated eyes compared with controls; however, we did not observe a consistent, treatment-specific hyphal deformation pattern that could be confidently assigned to the RZF or AFG groups (Fig. 6C). Therefore, Fig. 6C is interpreted primarily as supportive evidence of reduced intraocular fungal burden. Pathological scores were significantly lower in all treatment groups compared to the control, with no statistical difference between echinocandins (RZF, AFG) and the traditional drugs (L-AmB, VCZ) (Table 4; Fig. 6D).

**TABLE 4:** Histopathological scores

Groups	Histopathological scores
Control	11 ± 1
L-AmB	5.67 ± 1.53
VCZ	5.33 ± 0.58
RZF	4.33 ± 0.58
AFG	4.67 ± 0.58

ELISA quantification of aqueous TNF-α at Day 7 showed that all drug-treated groups exhibited significantly lower TNF-α levels than the control (Fig. 6E). Importantly, aqueous TNF-α in the L-AmB group was significantly lower than that in the VCZ, RZF, and AFG groups.

## DISCUSSION

Fungal endophthalmitis has garnered attention due to rising incidence, a high rate of blindness, and poor prognosis (16–18). Treatment challenges arise from the characteristics of fungal growth, the scarcity of effective antifungal drugs, and poor drug penetration into the eye. Current management includes surgical and pharmacological approaches; however, drug options for fungal endophthalmitis are limited, and the susceptibilities of different fungal species vary. Moreover, current clinical antifungals lack fungal cell specificity, affecting both fungal and host cells alike (19).

Amphotericin B (AmB) remains one of the most effective antifungals against *Candida* (20, 21). However, systemic administration causes significant dose-limiting side effects such as nephrotoxicity, and high-dose intraocular administration may induce severe inflammation and retinal damage (22). L-AmB, a lipid-formulated version of AmB, targets fungal cells to reduce host cell toxicity, thereby improving the therapeutic index. Although polyenes are historically associated with host toxicity, our study evaluated L-AmB, which has been repeatedly shown to be less toxic than conventional AmB deoxycholate in clinical use and experimental settings (15). Therefore, the higher *in vitro* IC50 values for L-AmB in mammalian ocular cell lines likely reflect the reduced bioavailability of free amphotericin and the established safety advantage of liposomal formulations. In contrast, while echinocandins target fungal β-glucan synthesis with no direct mammalian counterpart, supratherapeutic exposure and assay-dependent metabolic readouts may yield measurable effects on mammalian cells *in vitro* (23). With its broad-spectrum fungicidal action, it is regarded as a first-line option for fungal endophthalmitis (24). Our study confirmed that intravitreal injection of 10 μg L-AmB is both safe and effective at halting disease progression.

VCZ, a second-generation triazole, has broad activity against pathogens such as *Aspergillus*, *Candida*, and *Fusarium* and is widely used for fungal eye infections (25–28). Clinically, multiple intravitreal injections of VCZ have proven safe and effective, including in cases of *Candida* endophthalmitis resistant to fluconazole (29–31). Our *in vivo* and *in vitro* safety experiments also support that VCZ is well-tolerated in the eye. However, VCZ has limitations, including increasing azole resistance in non-*Candida albicans* species, such as *Candida glabrata* and *Candida krusei* (32). Another limitation is its short intraocular half-life (2.5–6.5 h) often requiring dosing every 2–3 days (33, 34). In addition, its metabolism via CYP3A4/5 poses a risk of drug interactions, necessitating therapeutic drug monitoring during systemic use (35, 36).

In summary, AmB and VCZ remain the cornerstones of ocular antifungal therapy. Newer echinocandins like RZF aim to combine broad fungicidal activity with improved safety and dosing convenience to address unmet needs in fungal endophthalmitis management. In our rabbit endophthalmitis model, L-AmB demonstrated a statistically significant advantage over VCZ, RZF, and AFG, yielding both the lowest residual intraocular CFU and the lowest aqueous TNF-α at Day 7. Beyond the intrinsic fungicidal activity of amphotericin B, the liposomal formulation itself may contribute to this superiority by altering pharmacokinetics and tissue distribution. Nevertheless, because intraocular drug concentrations were not measured in the present study, this mechanistic explanation remains speculative and warrants confirmation in future PK/PD studies. Despite these advantages, the need for safer and longer-acting systemic options remains, which motivates the evaluation of next-generation echinocandins such as RZF.

Echinocandin antifungals kill fungi by inhibiting β-(1, 3)-D-glucan synthase, thereby disrupting the fungal cell wall and causing osmotic imbalance and fungal lysis (8). Accordingly, echinocandins are generally associated with a favorable host safety profile because their primary target (β-(1, 3)-D-glucan synthase) is absent in mammalian cells (37). Echinocandins demonstrate potent activity against most *Candida* species and are increasingly recommended as first-line treatment for invasive candidiasis due to their efficacy and safety (6, 38, 39).

RZF, a novel second-generation echinocandin, possesses enhanced pharmacokinetic properties and potent activity against *Pneumocystis*, *Aspergillus*, and *Candida* (including azole-resistant strains) (40). It has been reported to show significant anti-biofilm effects against both adherent and mature *C. albicans* biofilms (12). RZF maintains broad fungicidal activity against *Candida* species, including azole-resistant strains such as *Candida glabrata* and *Candida auris* (41). These properties make RZF particularly valuable in ocular infections caused by strains with reduced azole susceptibility, such as emerging *C. auris* endophthalmitis (42, 43). Therefore, the antimicrobial spectrum and efficacy of RZF make it an important addition to the therapeutic armamentarium.

In this study, both AFG and the novel echinocandin RZF demonstrated significant therapeutic efficacy in treating rabbit *C. albicans* endophthalmitis, achieving outcomes comparable to standard agents L-AmB and VCZ. These findings support echinocandins as promising alternatives or adjuncts in clinical practice. Our results confirm and expand upon prior work by Karagoz *et al*., which first compared echinocandin (AFG, 50 μg) with AmB and VCZ in an experimental *C. albicans* endophthalmitis model (9). Our study extends this conclusion by introducing the next-generation echinocandin RZF: a single intravitreal injection of RZF (35 μg) or AFG (35 μg) achieved complete or near-complete clearance of intraocular *C. albicans*. Although minor differences in the speed of clinical improvement were observed, treatment outcomes converged across all groups by the end of one week. Thus, this suggests that we may have optimized the intravitreal dose of AFG—and by extension, RZF—reducing drug burden while maintaining efficacy.

Notably, RZF and AFG exhibited significantly lower MICs against *C. albicans* than L-AmB and VCZ, consistent with their rapid *in vivo* fungicidal activity. Because the assay used YPD at 30°C with an OD_600_ readout, the resulting MICs represent values obtained under our experimental conditions and are not directly interchangeable with clinical CLSI MICs; however, all drugs were tested side-by-side under identical conditions, enabling robust relative comparisons of antifungal potency. This suggests that future studies could explore lower therapeutic doses or longer dosing intervals, although pharmacokinetic and dose-response studies are needed to determine the minimum effective intraocular concentration.

At the microscopic level, we used SEM/TEM as a qualitative adjunct to visualize cellular injury after antifungal exposure. However, we acknowledge that several ultrastructural features (e.g., surface collapse and cytoplasmic disorganization) may represent common end-stage phenotypes of dead/dying cells irrespective of the killing method; therefore, Fig. 4A should be interpreted as supportive rather than diagnostic mechanistic evidence. Within this framework, the observed envelope-associated alterations are broadly consistent with established pharmacology: echinocandins can elicit compensatory cell-wall remodeling and associated structural changes (44); azole exposure has been reported to cause cell-wall thinning and membrane degradation (45); and for polyenes, while amphotericin B has historically been discussed in terms of pore-mediated membrane permeabilization, accumulating evidence supports that its primary fungicidal activity is better explained by self-assembly into extramembranous “sterol sponge” aggregates that extract ergosterol from membranes (46). Future work incorporating parallel non-drug killing controls (e.g., heat-inactivated cells) and/or more direct biochemical readouts (e.g., cell-wall composition or membrane permeability/sterol assays) would further strengthen mechanistic attribution.

All four antifungals also inhibited *C. albicans* biofilm formation. *C. albicans* biofilms—structured communities of yeast, hyphae, and extracellular matrix—are highly resistant to most antifungal agents and contribute significantly to treatment failure, as fungi within biofilms can withstand antifungal drug concentrations many times higher than planktonic cells (47). Crystal violet assays showed that at 8× MIC, echinocandins and L-AmB strongly suppressed biofilm development, outperforming VCZ. These results are consistent with earlier studies, which demonstrated that echinocandins and L-AmB possess unique efficacy in inhibiting *Candida* biofilm formation, while azoles have inherent limitations (48). It has been suggested that echinocandins, due to their mode of action on fungal cell walls (which remain exposed even within biofilms), can penetrate the biofilm matrix and kill fungi. In contrast, azoles inhibit ergosterol synthesis in fungal membranes, to which dormant biofilm cells are less sensitive. L-AmB formulations may better target biofilms by improving drug delivery into the matrix (49, 50). We note that our *in vitro* design focused on prevention (drug present during biofilm development) and did not test eradication of preformed/mature biofilms, which is often more clinically relevant and more drug-tolerant (51). Future studies will evaluate these agents against established biofilms to more fully assess their anti-biofilm potential.

In endophthalmitis, the concept of biofilm differs slightly from that of catheter-associated biofilms. In *C. albicans* endophthalmitis, fungi typically form inflammatory lesions in the choroid/retina and vitreous, often described as “snowbank-like” or “cotton-ball-like” lesions. These can be regarded as biofilm-like aggregates of fungi that are difficult for drugs to penetrate. Our histopathological results support the potential of echinocandins to not only exert fungicidal activity but also inhibit *in vivo* biofilm formation in such contexts.

Moreover, RZF, as a chemically modified analog of AFG, was designed for greater stability and persistence, resulting in a longer duration of effect and improved safety (12, 52). The phase 2 STRIVE and phase 3 ReSTORE trials indicated that once-weekly RZF was non-inferior to daily caspofungin in patients with candidemia/invasive candidiasis (38). After demonstrating efficacy and safety in clinical trials, RZF was approved for the treatment of candidemia and invasive candidiasis in adults (14, 38, 53). It also shows favorable safety in special populations without dose adjustment (54). A clinical report of intravitreal caspofungin in humans demonstrated several cases of fungal endophthalmitis successfully treated with caspofungin without apparent toxicity (55, 56), and AFG (50 μg) effectively treated experimental rabbit endophthalmitis without retinal toxicity (9), a finding reiterated in later reviews (9, 57). Because RZF is chemically similar to AFG (and caspofungin) but more stable, we anticipated its safety profile to be at least comparable or superior. Encouragingly, our results confirmed that intravitreal RZF (35 μg) displayed excellent ocular biocompatibility, indistinguishable from L-AmB or VCZ. Although RZF and AFG showed lower IC50 values than L-AmB and VCZ in mammalian cell assays, these results should be interpreted alongside antifungal potency and *in vivo* exposure. In ARPE-19 cells, the cytotoxicity-to-MIC ratios were >250 for L-AmB, >4,000 for VCZ, and 960–3,125 for RZF or AFG. The injected solution concentrations were 100 μg/mL for L-AmB, 500 μg/mL for VCZ, and 350 μg/mL for RZF or AFG. Given a rabbit vitreous volume of about 1.5 mL, the estimated initial vitreal concentrations were approximately 6-7 μg/mL, 30–35 μg/mL, and 20–25 μg/mL, respectively. These exposure levels are consistent with the absence of retinal toxicity in our fundus examination and histology. Echinocandins inhibit fungal β-(1, 3)-D-glucan synthase, a target absent in mammalian cells, which is consistent with their generally favorable host safety profile (37). Kernt et al. reported no apparent toxicity of caspofungin in human RPE cells after 24 h exposure up to 75 μg/mL (58). This is broadly consistent with our *in vitro* RPE data. Moreover, intravitreal caspofungin showed a dose threshold in mice, with no ERG or histologic abnormalities at 0.41–4.1 μM but reduced ERG amplitudes and ganglion cell loss at 41 μM (59). Together with our *in vivo* fundus examination and histology findings (Fig. 2), these data help reconcile the lower absolute IC50 values observed *in vitro* with acceptable tolerability at therapeutically relevant intraocular exposure.

RZF exhibits a markedly prolonged plasma half-life, exceeding 80 h after the first dose and reaching approximately 150 h after the second or third dose, enabling once-weekly systemic dosing for invasive candidiasis (41). Notably, due to its long-acting characteristics, RZF has already been validated as a prophylactic agent in high-risk patients (such as those undergoing bone marrow transplantation) (14, 60, 61). Its high stability and prolonged persistence may translate into extended vitreous retention time. Although we did not measure the pharmacokinetics of RZF in the vitreous, previous studies have shown that it possesses greater chemical stability and resistance to hydrolysis/photolysis at 37°C compared with older echinocandins. In contrast to drugs with relatively short vitreous half-lives, such as VCZ, this feature may reduce the need for frequent repeated injections (62–64). Given the risks of repeated intravitreal injections—including retinal detachment, hemorrhage, and infection—as well as difficulties with patient compliance, this property is highly attractive.

In the short term (2 h), RAW 264.7*-C. albicans* co-culture assay, we used 40× MIC t40× MIC to MIC-normalized supra-MIC pressure during the brief stimulation period, noting that MIC endpoints defined over longer incubations may not reflect early dynamics (65). This exposure remained below the cytotoxic range for RAW 264.7 cells in our CCK-8 assay. Because antifungals were present throughout stimulation, the decreased TNF-α and IL-1β transcripts reflect a net outcome under co-treatment; future work using dose-response and washout designs will help separate reduced fungal stimulation from potential host-directed immunomodulation.

The positive outcomes of this study have multiple implications for clinical practice and future research. *Candida* endophthalmitis most often occurs in patients with disseminated candidiasis (e.g., due to indwelling catheters, intravenous drug use, or immunosuppression) and is a vision-threatening condition. Up to one-third of patients with candidemia may develop ocular involvement, and if untreated, the prognosis is often poor (66). Current guidelines recommend combination therapy: systemic antifungal treatment (such as intravenous fluconazole or echinocandins) combined with early vitrectomy and intravitreal injection of L-AmB (5–10 μg) or VCZ (100 μg) for cases with significant vitreous involvement (39, 67). However, vitrectomy poses risks in critically ill patients, highlighting the need for non-surgical pharmacological strategies. Our findings suggest that intravitreal injection of echinocandins may represent a transformative option in this context. In particular, RZF offers the possibility of concurrent systemic and intraocular therapy, thereby simplifying treatment. For instance, an ICU patient with endogenous *C. albicans* endophthalmitis could receive a single intravitreal RZF injection alongside weekly intravenous RZF. Such a single-agent strategy may reduce the need for multiple drugs and their associated toxicities or interactions. In our rabbit experiments, a single intravitreal injection of RZF resulted in near-complete resolution of infection within one week. Furthermore, RZF may provide an alternative for patients unable to tolerate vitrectomy or those infected with resistant strains showing reduced susceptibility to existing therapies.

More importantly, RZF has not previously been systematically applied to ocular infections; this is the first evidence, based on a comprehensive evaluation system (cellular, animal, inflammatory, and morphological), to validate its feasibility for intraocular therapy. These findings align with expectations that echinocandins can effectively treat *Candida* endophthalmitis, representing a milestone in this field.

Although this study evaluated the candidacy of RZF from multiple perspectives, several limitations remain. First, intraocular pharmacokinetic studies in humans or non-human primates are lacking. Second, we used an exogenous intravitreal inoculation model to enable controlled assessment of intravitreal therapy; future studies should validate RZF/AFG in a hematogenous intravenous model of endogenous *Candida* endophthalmitis to strengthen clinical generalizability. Moreover, our model employed a single injection after infection was established; future studies should explore multiple dosing regimens, drug combinations, or combination with surgical interventions, which may be beneficial for more severe or refractory cases. Additionally, we only investigated a single standard strain of *C. albicans*. Variations in virulence among different strains may influence drug efficacy, and further research should extend to other pathogenic fungi, such as filamentous fungi. The product is not labeled for the use under discussion.

### Conclusion

In summary, our study was the first to comprehensively evaluate the value of a new-generation echinocandin in an ocular *Candida* infection model. In the rabbit model of *C. albicans* endophthalmitis, RZF proved to be a potent and safe antifungal agent, with efficacy comparable to the current standard treatments (L-AmB and VCZ), and it demonstrated strong *in vitro* inhibition of *C. albicans* biofilm formation under our experimental conditions. AFG similarly reinforces the view that echinocandins are effective in the eye, confirming previous findings and achieving this at an optimized dose. These results support the notion that echinocandins, especially RZF, could be used as a first-line intravitreal therapy for fungal endophthalmitis. The clinical introduction of RZF can expand our therapeutic arsenal, providing an alternative for patients who cannot tolerate conventional therapy or whose infecting strains have reduced susceptibility to existing drugs. Moreover, RZF’s unique pharmacologic properties (long half-life and stability) may translate into practical benefits such as fewer injections and simplified systemic therapy.

Finally, although L-AmB and VCZ remain important therapeutic tools, the emergence of drugs like RZF represents a significant advance in antifungal therapy. Successful application of RZF in *C. albicans* endophthalmitis will exemplify how innovations in systemic antifungal drug development can be translated to address longstanding challenges in ophthalmology.

## MATERIALS AND METHODS

### Cell culture

ARPE-19 human retinal pigment epithelial cells and human Müller glial cells were obtained from the Cell Bank of the Chinese Academy of Sciences (Shanghai, China). RAW 264.7 murine macrophages were obtained from China National Pharmaceutical & Biological Products (Beijing, China). ARPE-19 and Müller cells were maintained in RPMI 1640 medium, and RAW 264.7 cells were maintained in DMEM, with each medium supplemented with 10% fetal bovine serum and 1% penicillin-streptomycin. Cells were incubated at 37°C in 5% CO₂.

### Cell compatibility evaluation

The cytotoxicity of the four drugs was evaluated using ARPE-19, Müller, and RAW 264.7 cells. Cells were seeded in 96-well plates at densities of 1 × 10⁴, 3 × 10⁴, and 3 × 10⁴ cells per well, respectively, and allowed to adhere. The cells were exposed to varying drug concentrations for 24 h. Cell viability was measured using the CCK-8 assay (Beyotime Biotechnology, Shanghai, China). Each cell type was plated at 1 × 10⁴ cells per well in 96-well plates; after adherence, cells were treated for 72 h with the highest drug concentration that allowed for ≥90% cell viability (determined from a CCK-8 pre-test). Live/dead ratios were determined using Calcein-AM/PI double staining (Beyotime).

### *In vivo* biocompatibility evaluation

Five healthy adult male rabbits were randomly divided into five groups (*n* = 1/ group): intravitreal saline (0.1 mL), L-AmB (10 μg/0.1 mL), VCZ (50 μg/0.1 mL), RZF (35 μg/0.1 mL), or AFG (35 μg/0.1 mL). After 3 days, color fundus photographs were taken, and the rabbits were then euthanized. The eyes, liver, spleen, and kidneys were harvested and fixed in 4% paraformaldehyde at 4°C for 24 h. Tissues were processed through graded ethanol, xylene, and paraffin and then sectioned at 5 μm. Each tissue was stained with hematoxylin-eosin (H&E), and ocular sections were additionally stained with Masson’s trichrome. Histopathological changes were observed under a light microscope.

### *C. albicans* culture

Yeast extract peptone dextrose (YPD, Hope Bio-Technology Co., Ltd, Qingdao, China) agar plates were prepared by dissolving 30 g of YPD medium and 8 g of agar in 600 mL of pure water, autoclaving at 121°C for 15 min, pouring into 70 mm-diameter plates, and solidifying. YPD liquid media were similarly prepared without agar. The standard *C. albicans* strain (ATCC 10231) was streaked onto a YPD plate using a sterile loop and incubated inverted at 30°C for 48 h.

### Minimum inhibitory concentration determination

The MICs were determined by a broth microdilution assay modified from CLSI M27 (4th ed.) for the experimental conditions used in this study (68). Briefly, *C. albicans* was prepared at 2 × 10³ CFU/mL in YPD medium and dispensed into 96-well plates containing serially diluted antifungal agents. Plates were incubated at 30°C for 48 h, and growth was quantified by OD₆₀₀. MIC endpoints were defined as ≥50% growth inhibition relative to the drug-free control for VCZ and echinocandins, and complete inhibition for L-AmB.

### Biofilm inhibition assay

Biofilm formation by *C. albicans* was assessed in 96-well plates using crystal violet staining. Briefly, 100 μL of a *C. albicans* suspension (2 × 10³ CFU/mL) was added to each well, followed by 100 μL of drug solution at 1×, 2×, 4×, or 8× MIC. After 48 h of incubation at 30°C, the culture was gently removed. Wells were washed with sterile PBS, fixed with methanol, stained with 0.1% crystal violet, rinsed, dried, and decolorized with 33% acetic acid. The stained biofilms were photographed, and the OD of each well was measured at 570 nm for statistical analysis.

### Fungal live/dead staining assay

To visualize drug-induced damage to fungal cells, a live/dead staining assay was performed on biofilms grown on coverslips. In a 24-well plate containing sterile coverslips, 1 mL of *C. albicans* (2 × 10⁶ CFU/mL) and 1 mL of each drug at 4× MIC were added to each well and incubated at 30°C for 48 h. After removal of supernatants and gentle washing, biofilms on the coverslips were stained with DMAO and propidium iodide (PI) in the dark and incubated at room temperature for 20 min. The coverslips were then mounted on microscope slides, air-dried, and imaged under a confocal laser scanning microscope (CLSM). Both DMAO and PI are fluorescent nucleic acid dyes. DMAO stains both live and dead fungi green, whereas PI stains necrotic fungi red.

### Ultrastructural fungal morphology observation

Fungal ultrastructure after drug treatment was examined by electron microscopy. *C. albicans* cells (2 × 10⁶ CFU/mL) were incubated with L-AmB (16 μg/mL), VCZ (1.0 μg/mL), RZF (0.125 μg/mL), or AFG (0.125 μg/mL) (4× MIC) for 48 h at 30°C, harvested by centrifugation, and fixed in 2.5% glutaraldehyde. After fixation, samples were dehydrated through a graded ethanol series, critical-point dried, and platinum-sputtered. The treated specimens were examined using a field-emission scanning electron microscope (SEM) and a transmission electron microscope (TEM).

### Macrophage cytokine mRNA measurement (qRT-PCR)

Based on the method of Chryssanthou *et al*. with modifications (65), RAW 264.7 cells (3 × 10^6^ cells/well) were seeded in 6-well plates and adhered overnight. Cells were then stimulated with *C. albicans* (3 × 10^7^ CFU/mL) and treated for 2 h at 37°C with L-AmB (160 μg/mL), VCZ (10 μg/mL), RZF (1.25 μg/mL), or AFG (1.25 μg/mL), corresponding to 40× the MIC values determined in this study. After stimulation, cells were harvested, and total RNA was extracted using the RNAeasy Animal RNA Isolation Kit (Beyotime). One-step qRT-PCR was performed using a BeyoFast SYBR Green protocol on a LightCycler 96 thermocycler. The primer sequences for TNF-α, IL-1β, and the reference gene (GAPDH) are listed in Table S1 (69). Cycling conditions consisted of cDNA synthesis at 50°C for 15 min, initial denaturation at 95°C for 2 min, and 40 cycles of 95°C for 15 s and 60°C for 30 s. Relative mRNA expression levels were calculated by the 2^−ΔΔCt^ method.

### Rabbit endophthalmitis model establishment

Twenty-five healthy adult male rabbits (1.4–1.6 kg) were used to establish a *C. albicans* endophthalmitis model. Under isoflurane anesthesia, each rabbit received an intravitreal injection of 100 μL *C. albicans* (4.0 × 10^6^ CFU/mL) in the right eye. At 48 h post-infection (Day 0), the rabbits were randomly assigned to five groups (*n* = 5): Control (0.1 mL normal saline, NS), L-AmB (10 μg/0.1 mL), VCZ (50 μg/0.1 mL), RZF (35 μg/0.1 mL), and AFG (35 μg/0.1 mL). A single intravitreal injection was administered at this time point to assess therapeutic efficacy.

### Endophthalmitis clinical scoring

External examination and photography were performed at each time point. Day 0 assessments (clinical scoring and images) were completed immediately prior to intravitreal injection at 48 h post-infection, and follow-up assessments were performed on Days 1, 3, 5, and 7. The severity of intraocular inflammation was scored according to the grading criteria in Table S2 (9). Scores at each time point were recorded and statistically analyzed.

### Aqueous humor cytokine measurement

On Day 7, an anterior chamber paracentesis was performed on each infected eye to collect aqueous humor before euthanasia. The concentration of TNF-α in the aqueous humor of each eye was determined using a quantitative ELISA kit (Beyotime). The same infected eye was subsequently used for terminal sampling (either CFU or histology) according to the random allocation described above.

### Endophthalmitis antifungal efficacy evaluation

On Day 7, after euthanasia, infected eyes allocated for fungal burden analysis (*n* = 3 eyes/group) were enucleated and processed for CFU quantification immediately. Ocular tissues were aseptically minced and homogenized at 4°C, serially diluted in NS, and spread onto YPD agar plates. After incubating inverted at 30°C for 48 h, fungal colonies were counted. The fungal burden in each eye was expressed as CFU, and the fungicidal rate was calculated as follows: Fungicidal Rate = (1 − CFU of treatment group/CFU of control group) × 100%.

### Histological analysis

On Day 7 post-euthanasia, infected eyes allocated for histological analysis (*n* = 2 eyes/group) were enucleated and fixed. Ocular tissue sections were stained with H&E, periodic acid–Schiff (PAS), and Masson’s trichrome to evaluate tissue morphology and the extent of fungal infiltration. Inflammation and damage were scored based on Table S3 (70) and statistically analyzed.

### Statistical analysis

The experimental data were analyzed using GraphPad Prism 8. One-way analysis of variance (ANOVA) was used for multiple-group comparisons. When ANOVA indicated significance, Tukey’s multiple-comparisons test was performed for post-hoc pairwise comparisons among groups. For *in vitro* assays, data are presented as mean ± SD from three independent experiments, with ≥3 technical replicates per condition per experiment. Microscopy images are representative of ≥3 independent experiments. For the *in vivo* rabbit efficacy study, the experiment was conducted once as a single cohort, and n denotes the number of eyes analyzed per group for each endpoint. *P < 0.05; **P < 0.01; ***P < 0.001.

## SUPPLEMENTAL MATERIAL

Supplemental material aac.01670-25-s0001.docx Fig. S1; Tables S1 to S3.

ASM does not own the copyrights to Supplemental Material that may be linked to, or accessed through, an article. The authors have granted ASM a non-exclusive, world-wide license to publish the Supplemental Material files. Please contact the corresponding author directly for reuse.

## References

[1] Bihaniya H, Rudraprasad D, Joseph J. 2024. Pathobiology of fungal endophthalmitis: a major review. ACS Infect Dis 10:3126–3137. doi:10.1021/acsinfecdis.4c00442 39267469

[2] Durand ML. 2013. Endophthalmitis. Clin Microbiol Infect 19:227–234. doi:10.1111/1469-0691.12118 23438028 PMC3638360

[3] Patil A, Majumdar S. 2017. Echinocandins in ocular therapeutics. J Ocul Pharmacol Ther 33:340–352. doi:10.1089/jop.2016.0186 28437176

[4] Khambati A, Wright RE III, Das S, Pasula S, Sepulveda A, Hernandez F, Kanwar M, Chandrasekar P, Kumar A. 2022. Aspergillus endophthalmitis: epidemiology, pathobiology, and current treatments. J Fungi (Basel) 8:656. doi:10.3390/jof8070656 35887412 PMC9318612

[5] Radhika M, Mithal K, Bawdekar A, Dave V, Jindal A, Relhan N, Albini T, Pathengay A, Flynn HW. 2014. Pharmacokinetics of intravitreal antibiotics in endophthalmitis. J Ophthalmic Inflamm Infect 4:22. doi:10.1186/s12348-014-0022-z 25667683 PMC4306439

[6] Salazar SB, Simões RS, Pedro NA, Pinheiro MJ, Carvalho MFNN, Mira NP. 2020. An overview on conventional and non-conventional therapeutic approaches for the treatment of candidiasis and underlying resistance mechanisms in clinical strains. J Fungi (Basel) 6:23. doi:10.3390/jof6010023 32050673 PMC7151124

[7] Lohse MB, Gulati M, Johnson AD, Nobile CJ. 2018. Development and regulation of single- and multi-species Candida albicans biofilms. Nat Rev Microbiol 16:19–31. doi:10.1038/nrmicro.2017.107 29062072 PMC5726514

[8] Fesel PH, Zuccaro A. 2016. β-glucan: crucial component of the fungal cell wall and elusive MAMP in plants. Fungal Genet Biol 90:53–60. doi:10.1016/j.fgb.2015.12.004 26688467

[9] Karagoz E, Ugan RA, Duzgun E, Cadirci E, Keles S, Uyanik MH, Yavan I, Turhan V. 2017. A comparative study of the effects of intravitreal anidulafungin, voriconazole, and amphotericin B in an experimental candida endophthalmitis model. Curr Eye Res 42:225–232. doi:10.3109/02713683.2016.1170857 27348425

[10] Garcia-Effron G. 2020. Rezafungin-mechanisms of action, susceptibility and resistance: similarities and differences with the other echinocandins. J Fungi (Basel) 6:262. doi:10.3390/jof6040262 33139650 PMC7711656

[11] Cota JM, Grabinski JL, Talbert RL, Burgess DS, Rogers PD, Edlind TD, Wiederhold NP. 2008. Increases in SLT2 expression and chitin content are associated with incomplete killing of Candida glabrata by caspofungin. Antimicrob Agents Chemother 52:1144–1146. doi:10.1128/AAC.01542-07 18086838 PMC2258485

[12] Chandra J, Ghannoum MA. 2018. CD101, a novel echinocandin, possesses potent antibiofilm activity against early and mature Candida albicans biofilms. Antimicrob Agents Chemother 62:e01750-17. doi:10.1128/AAC.01750-17 29133552 PMC5786756

[13] Thompson GR III, Soriano A, Cornely OA, Kullberg BJ, Kollef M, Vazquez J, Honore PM, Bassetti M, Pullman J, Chayakulkeeree M, et al.. 2023. Rezafungin versus caspofungin for treatment of candidaemia and invasive candidiasis (ReSTORE): a multicentre, double-blind, double-dummy, randomised phase 3 trial. The Lancet 401:49–59. doi:10.1016/S0140-6736(22)02324-8 36442484

[14] Syed YY. 2023. Rezafungin: first approval. Drugs (Abingdon Engl) 83:833–840. doi:10.1007/s40265-023-01891-8 37212966

[15] Stone NRH, Bicanic T, Salim R, Hope W. 2016. Liposomal Amphotericin B (AmBisome): a review of the pharmacokinetics, pharmacodynamics, clinical experience and future directions. Drugs (Abingdon Engl) 76:485–500. doi:10.1007/s40265-016-0538-7 PMC4856207 26818726

[16] Li X, Chen Z, Zhang X, Zhou Z, Boost M, Huang T, Zhou X. 2024. Fungal endophthalmitis: clinical characteristics, pathogens, and factors affecting visual outcome. Antibiotics (Basel) 13:199. doi:10.3390/antibiotics13030199 38534634 PMC10967284

[17] Dedieu D, Contejean A, Gastli N, Marty-Reboul J, Poupet H, Brezin A, Monnet D, Charlier C, Canouï E. 2024. Endogenous endophthalmitis: new insights from a 12-year cohort study. Int J Infect Dis 146:107116. doi:10.1016/j.ijid.2024.107116 38801969

[18] Kim SW, Kim JH, Choi M, Lee SJ, Shin JP, Kim JG, Kang SW, Park KH, Members KRS, Nam DH, et al.. 2023. An outbreak of fungal endophthalmitis after cataract surgery in South Korea. JAMA Ophthalmol 141:226–233. doi:10.1001/jamaophthalmol.2022.5927 36656597 PMC9857837

[19] Ambati S, Pham T, Lewis ZA, Lin X, Meagher RB. 2022. Dectisomes: glycan targeting of liposomal drugs improves the treatment of disseminated candidiasis. Antimicrob Agents Chemother 66:e0146721. doi:10.1128/AAC.01467-21 34633846 PMC8765427

[20] Lewis RE, Kontoyiannis DP, Darouiche RO, Raad II, Prince RA. 2002. Antifungal activity of amphotericin B, fluconazole, and voriconazole in an in vitro model of Candida catheter-related bloodstream infection. Antimicrob Agents Chemother 46:3499–3505. doi:10.1128/AAC.46.11.3499-3505.2002 12384356 PMC128760

[21] Al Jalali V, Sauermann R, Eberl S, Zeitlinger M. 2019. In vitro activity of voriconazole and amphotericin B against Candida albicans, Candida krusei, and Cryptococcus neoformans in human cerebrospinal fluid. Infection 47:565–570. doi:10.1007/s15010-019-01275-9 30725316

[22] Cannon JP, Fiscella R, Pattharachayakul S, Garey KW, De Alba F, Piscitelli S, Edward DP, Danziger LH. 2003. Comparative toxicity and concentrations of intravitreal amphotericin B formulations in a rabbit model. Invest Ophthalmol Vis Sci 44:2112–2117. doi:10.1167/iovs.02-1020 12714650

[23] Martinez LR, Ntiamoah P, Casadevall A, Nosanchuk JD. 2007. Caspofungin reduces the incidence of fungal contamination in cell culture. Mycopathologia 164:279–286. doi:10.1007/s11046-007-9063-2 17899440

[24] Zhao J, Cheng Y, Song X, Wang C, Su G, Liu Z. 2015. A comparative treatment study of intravitreal voriconazole and liposomal amphotericin B in an aspergillus fumigatus endophthalmitis model. Invest Ophthalmol Vis Sci 56:7369–7376. doi:10.1167/iovs.15-17266 26574795

[25] Marangon FB, Miller D, Giaconi JA, Alfonso EC. 2004. In vitro investigation of voriconazole susceptibility for keratitis and endophthalmitis fungal pathogens. Am J Ophthalmol 137:820–825. doi:10.1016/j.ajo.2003.11.078 15126145

[26] Messer SA, Carvalhaes CG, Castanheira M, Pfaller MA. 2020. In vitro activity of isavuconazole versus opportunistic filamentous fungal pathogens from the SENTRY Antifungal Surveillance Program, 2017-2018. Diagn Microbiol Infect Dis 97:115007. doi:10.1016/j.diagmicrobio.2020.115007 32081523

[27] Guinea J, Peláez T, Recio S, Torres-Narbona M, Bouza E. 2008. In vitro antifungal activities of isavuconazole (BAL4815), voriconazole, and fluconazole against 1,007 isolates of zygomycete, Candida, Aspergillus, Fusarium, and Scedosporium species. Antimicrob Agents Chemother 52:1396–1400. doi:10.1128/AAC.01512-07 18212101 PMC2292541

[28] Pfaller MA, Rhomberg PR, Wiederhold NP, Gibas C, Sanders C, Fan H, Mele J, Kovanda LL, Castanheira M. 2018. In vitro activity of isavuconazole against opportunistic fungal pathogens from two mycology reference laboratories. Antimicrob Agents Chemother 62:e01230-18. doi:10.1128/AAC.01230-18 30061288 PMC6153788

[29] Bienvenu A-L, Aussedat M, Mathis T, Guillaud M, Leboucher G, Kodjikian L. 2020. Intravitreal injections of voriconazole for Candida endophthalmitis: a case series. Ocul Immunol Inflamm 28:471–478. doi:10.1080/09273948.2019.1571613 30810429

[30] Xie Y, Wang X, Ji Z, Li G, Zhang C. 2024. The effectiveness and safety of intravitreal injections of voriconazole in the treatment of fungal endophthalmitis: a systematic review. J Ocul Pharmacol Ther 40:332–341. doi:10.1089/jop.2023.0103 38011696

[31] Danielescu C, Anton N, Stanca HT, Munteanu M. 2020. Endogenous endophthalmitis: a review of case series published between 2011 and 2020. J Ophthalmol 2020:8869590. doi:10.1155/2020/8869590 33149945 PMC7603614

[32] Ajetunmobi OH, Badali H, Romo JA, Ramage G, Lopez-Ribot JL. 2023. Antifungal therapy of Candida biofilms: past, present and future. Biofilm 5:100126. doi:10.1016/j.bioflm.2023.100126 37193227 PMC10182175

[33] Shen Y-C, Wang M-Y, Wang C-Y, Tsai T-C, Tsai H-Y, Lee H-N, Wei L-C. 2009. Pharmacokinetics of intracameral voriconazole injection. Antimicrob Agents Chemother 53:2156–2157. doi:10.1128/AAC.01125-08 19258273 PMC2681505

[34] Das T, Joseph J, Jakati S, Sharma S, Velpandian T, Padhy SK, Das VA, Shivaji S, Nayak S, Behera UC, Mishra DK, Gandhi J, Dave VP, Pathengay A. 2022. Understanding the science of fungal endophthalmitis - AIOS 2021 sengamedu srinivas badrinath endowment lecture. Indian J Ophthalmol 70:768–777. doi:10.4103/ijo.IJO_2329_21 35225510 PMC9114621

[35] Clary RT, Deja E, Rittmann B, Bearman G. 2025. Impact of voriconazole therapeutic drug monitoring on adverse effects and clinical outcomes: a literature review. Curr Infect Dis Rep 27:6. doi:10.1007/s11908-025-00856-0

[36] Champagne W, Malek AE. 2025. Cytochrome P450 genotype polymorphisms and suboptimal voriconazole serum level in a patient with invasive cerebral fungal infection. IDCases 41:e02332. doi:10.1016/j.idcr.2025.e02332 40762011 PMC12320098

[37] Perlin DS. 2011. Current perspectives on echinocandin class drugs. Future Microbiol 6:441–457. doi:10.2217/fmb.11.19 21526945 PMC3913534

[38] Thompson GR, Soriano A, Honore PM, Bassetti M, Cornely OA, Kollef M, Kullberg BJ, Pullman J, Hites M, Fortún J, Horcajada JP, Kotanidou A, Das AF, Sandison T, Aram JA, Vazquez JA, Pappas PG. 2024. Efficacy and safety of rezafungin and caspofungin in candidaemia and invasive candidiasis: pooled data from two prospective randomised controlled trials. Lancet Infect Dis 24:319–328. doi:10.1016/S1473-3099(23)00551-0 38008099

[39] Pappas PG, Kauffman CA, Andes DR, Clancy CJ, Marr KA, Ostrosky-Zeichner L, Reboli AC, Schuster MG, Vazquez JA, Walsh TJ, Zaoutis TE, Sobel JD. 2016. Clinical practice guideline for the management of candidiasis: 2016 update by the infectious diseases society of America. Clin Infect Dis 62:e1–50. doi:10.1093/cid/civ933 26679628 PMC4725385

[40] Ordaya EE, Clement J, Vergidis P. 2023. The role of novel antifungals in the management of candidiasis: a clinical perspective. Mycopathologia 188:937–948. doi:10.1007/s11046-023-00759-5 37470902 PMC10687117

[41] Sandison T, Ong V, Lee J, Thye D. 2017. Safety and pharmacokinetics of CD101 IV, a novel echinocandin, in healthy adults. Antimicrob Agents Chemother 61:e01627-16. doi:10.1128/AAC.01627-16 27919901 PMC5278714

[42] Locke JB, Andes D, Flanagan S, Redell M, Ong V, Aram JA, Pappas PG, Castanheira M, Thompson GR. 2025. Activity of rezafungin against Candida auris. J Antimicrob Chemother 80:1482–1493. doi:10.1093/jac/dkaf124 40304092 PMC12129586

[43] Oliva A, De Rosa FG, Mikulska M, Pea F, Sanguinetti M, Tascini C, Venditti M. 2023. Invasive Candida infection: epidemiology, clinical and therapeutic aspects of an evolving disease and the role of rezafungin. Expert Rev Anti Infect Ther 21:957–975. doi:10.1080/14787210.2023.2240956 37494128

[44] Walker LA, Munro CA. 2020. Caspofungin induced cell wall changes of Candida species influences macrophage interactions. Front Cell Infect Microbiol 10:164. doi:10.3389/fcimb.2020.00164 32528900 PMC7247809

[45] Sorgo AG, Heilmann CJ, Dekker HL, Bekker M, Brul S, de Koster CG, de Koning LJ, Klis FM. 2011. Effects of fluconazole on the secretome, the wall proteome, and wall integrity of the clinical fungus Candida albicans. Eukaryot Cell 10:1071–1081. doi:10.1128/EC.05011-11 21622905 PMC3165447

[46] Maji A, Soutar CP, Zhang J, Lewandowska A, Uno BE, Yan S, Shelke Y, Murhade G, Nimerovsky E, Borcik CG, et al.. 2023. Tuning sterol extraction kinetics yields a renal-sparing polyene antifungal. Nature 623:1079–1085. doi:10.1038/s41586-023-06710-4 37938782 PMC10883201

[47] Roscetto E, Contursi P, Vollaro A, Fusco S, Notomista E, Catania MR. 2018. Antifungal and anti-biofilm activity of the first cryptic antimicrobial peptide from an archaeal protein against Candida spp. clinical isolates. Sci Rep 8:17570. doi:10.1038/s41598-018-35530-0 30514888 PMC6279838

[48] Kuhn DM, George T, Chandra J, Mukherjee PK, Ghannoum MA. 2002. Antifungal susceptibility of Candida biofilms: unique efficacy of amphotericin B lipid formulations and echinocandins. Antimicrob Agents Chemother 46:1773–1780. doi:10.1128/AAC.46.6.1773-1780.2002 12019089 PMC127206

[49] Marcos-Zambrano LJ, Gómez-Perosanz M, Escribano P, Zaragoza O, Bouza E, Guinea J. 2016. Biofilm production and antibiofilm activity of echinocandins and liposomal amphotericin B in echinocandin-resistant yeast species. Antimicrob Agents Chemother 60:3579–3586. doi:10.1128/AAC.03065-15 27021323 PMC4879372

[50] Tits J, Cammue BPA, Thevissen K. 2020. Combination therapy to treat fungal biofilm-based infections. Int J Mol Sci 21:8873. doi:10.3390/ijms21228873 33238622 PMC7700406

[51] Ferreira JAG, Carr JH, Starling CEF, de Resende MA, Donlan RM. 2009. Biofilm formation and effect of caspofungin on biofilm structure of Candida species bloodstream isolates. Antimicrob Agents Chemother 53:4377–4384. doi:10.1128/AAC.00316-09 19546368 PMC2764222

[52] Ong V, Hough G, Schlosser M, Bartizal K, Balkovec JM, James KD, Krishnan BR. 2016. Preclinical evaluation of the stability, safety, and efficacy of CD101, a novel echinocandin. Antimicrob Agents Chemother 60:6872–6879. doi:10.1128/AAC.00701-16 27620474 PMC5075098

[53] Ponta G, Morena V, Strano M, Molteni C, Pontiggia S, Cavalli EM, Grancini A, Mauri C, Castagna A, Galanti A, Piconi S. 2024. Safety of rezafungin as a long-term treatment option in two patients with complicated fungal infections: two cases from Lecco Hospital (Italy). Antimicrob Agents Chemother 68. doi:10.1128/aac.00750-24 PMC11304680 38995032

[54] Cornely OA, Dupont H, Mikulska M, Rautemaa-Richardson R, Garcia-Vidal C, Thompson GR, Hoenigl M. 2025. Rezafungin in special populations with candidaemia and/or invasive candidiasis. J Infect 90:106435. doi:10.1016/j.jinf.2025.106435 39921063

[55] Garrido-Marin M, Kirkegaard Biosca E, Boixadera A, Fischer Fernandez R, Sánchez Vela L, Pardo Aranda A, García-Arumí J, Distefano L. 2024. Multiresistant Candida endophthalmitis treated with intravitreal caspofungin: a case report. Ocul Immunol Inflamm 32:858–862. doi:10.1080/09273948.2023.2168701 36696576

[56] von Jagow B, Kurzai O, Kakkassery V. 2020. Case report: beyond the blood-retina barrier: intravitreal caspofungin for fungal endophthalmitis. Optom Vis Sci 97:473–476. doi:10.1097/OPX.0000000000001532 32697551

[57] Reginatto P, Agostinetto G de J, Fuentefria R do N, Marinho DR, Pizzol MD, Fuentefria AM. 2023. Eye fungal infections: a mini review. Arch Microbiol 205:236. doi:10.1007/s00203-023-03536-6 37183227 PMC10183313

[58] Kernt M, Kampik A. 2011. Intraocular caspofungin: in vitro safety profile for human ocular cells. Mycoses 54:e110–21. doi:10.1111/j.1439-0507.2009.01853.x 20202116

[59] Mojumder DK, Concepcion FA, Patel SK, Barkmeier AJ, Carvounis PE, Wilson JH, Holz ER, Wensel TG. 2010. Evaluating retinal toxicity of intravitreal caspofungin in the mouse eye. Invest Ophthalmol Vis Sci 51:5796. doi:10.1167/iovs.10-5541 20505203 PMC2932784

[60] Albanell-Fernández M. 2025. Echinocandins pharmacokinetics: a comprehensive review of micafungin, caspofungin, anidulafungin, and rezafungin population pharmacokinetic models and dose optimization in special populations. Clin Pharmacokinet 64:27–52. doi:10.1007/s40262-024-01461-5 39707078 PMC11762474

[61] Brown J, Lakota EA, Flanagan S, Sandison T, Ong V, Rubino CM. 2019. Pharmacokinetic-pharmacodynamic analyses of dose selection for rezafungin prophylaxis against invasive fungal infections in bone marrow transplantation. Biol Blood Marrow Transplant 25:S358–S359. doi:10.1016/j.bbmt.2018.12.581

[62] Garcia-Effron G. 2020. Rezafungin—mechanisms of action, susceptibility and resistance: similarities and differences with the other echinocandins. J Fungi (Basel) 6:262. doi:10.3390/jof6040262 33139650 PMC7711656

[63] Ham YY, Lewis JS, Thompson GR. 2021. Rezafungin: a novel antifungal for the treatment of invasive candidiasis. Future Microbiol 16:27–36. doi:10.2217/fmb-2020-0217 33438477

[64] Shen Y-C, Wang M-Y, Wang C-Y, Tsai T-C, Tsai H-Y, Lee Y-F, Wei L-C. 2007. Clearance of intravitreal voriconazole. Invest Ophthalmol Vis Sci 48:2238–2241. doi:10.1167/iovs.06-1362 17460285

[65] Chryssanthou E, Loebig A, Sjölin J. 2008. Post-antifungal effect of amphotericin B and voriconazole against germinated Aspergillus fumigatus conidia. J Antimicrob Chemother 61:1309–1311. doi:10.1093/jac/dkn129 18367461

[66] Phongkhun K, Pothikamjorn T, Srisurapanont K, Manothummetha K, Sanguankeo A, Thongkam A, Chuleerarux N, Leksuwankun S, Meejun T, Thanakitcharu J, Walker M, Gopinath S, Torvorapanit P, Langsiri N, Worasilchai N, Moonla C, Plongla R, Kates OS, Nematollahi S, Permpalung N. 2023. Prevalence of ocular Candidiasis and Candida endophthalmitis in patients with candidemia: a systematic review and meta-analysis. Clin Infect Dis 76:1738–1749. doi:10.1093/cid/ciad064 36750934 PMC10411939

[67] Danielescu C, Stanca HT, Iorga R-E, Darabus D-M, Potop V. 2022. The diagnosis and treatment of fungal endophthalmitis: an update. Diagnostics (Basel) 12:679. doi:10.3390/diagnostics12030679 35328231 PMC8947249

[68] CLSI. 2017. Reference method for broth dilution antifungal susceptibility testing of yeasts. 4th ed. CLSI Standard M27. Clinical and Laboratory Standards Institute, Wayne, PA.

[69] Zhang T, Wang Y, Li R, Xin J, Zheng Z, Zhang X, Xiao C, Zhang S. 2023. ROS-responsive magnesium-containing microspheres for antioxidative treatment of intervertebral disc degeneration. Acta Biomater 158:475–492. doi:10.1016/j.actbio.2023.01.020 36640954

[70] Saleh M, Lefèvre S, Acar N, Bourcier T, Marcellin L, Prévost G, Subilia A, Gaucher D, Jehl F. 2012. Efficacy of intravitreal administrations of linezolid in an experimental model of S. aureus-related endophthalmitis. Invest Ophthalmol Vis Sci 53:4832–4841. doi:10.1167/iovs.11-8417 22661478

