# Supplementary material for: Safety and efficacy of echinocandin antifungal agents in Candida albicans endophthalmitis
Source: Antimicrob Agents Chemother. 2026 Mar 23;70(5):e01670-25. doi: 10.1128/aac.01670-25 (PMC13148047; doi:10.1128/aac.01670-25)
Supplement: Supplemental material — Fig. S1; Tables S1 to S3. [file aac.01670-25-s0001.docx]

**Title: Safety and Efficacy of Echinocandin Antifungal Agents in Candida albicans Endophthalmitis**

**Authors:**

Yue Zhang^1,2^, MS, Yi Tang^1^, MS, Yanjie Zhou^1^, MS, Hong Wu^1^, MD

1: Department of Ophthalmology, the Second Hospital of Jilin University, Changchun, Jilin Province, China. This research was funded by the Jilin Science and Technology Development Program Project (Grant No. 232670GH0101111521).

2: Department of Ophthalmology, Changzhi People's Hospital, Changzhi, Shanxi Province, China.

**Supplementary Materials**

**Supplementary Figure S1
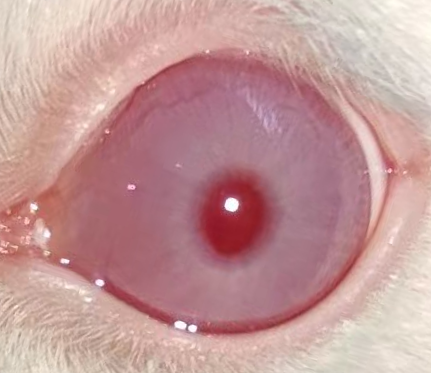
**

Representative external photograph of a healthy, uninfected rabbit eye.

**Supplementary Table S1**

Primer sequences used for qRT-PCR analysis.

| Gene | Sense primers (5'-3') | Antisense primers (5'-3') |
| --- | --- | --- |
| IL-1β | ATCAGGACAGCCCAGGTCAA | GCCACCTTTTGACAGTGATGAG |
| TNF-α | ACCCTCACACTCACAAACCA | GCAGCCTTGTCCCTTGAAGA |
| GAPDH | GCATCTTCTTGTGCAGTGCC | GGTAACCAGGCGTCCGATAC |

**Supplementary Table S2**

Clinical scoring scheme.

| Grade | Cornea | Conjunctiva | Iris | Vitreous |
| --- | --- | --- | --- | --- |
| 0 | Clear | Normal | Normal | Clear |
| 1 | Focal edema | Mild edema | Mild hyperemia | Areas of vitreous haze, some fundus details visible, good red reflex |
| 2 | Diffuse edema | Edema, mild hyperemia, slight exudate | Marked hyperemia | Moderate vitreous haze, no fundus details visible, partial red reflex |
| 3 | Opaque | Edema, marked hyperemia, heavy exudate | Marked hyperemia, synechiae, irregular pupil | No red reflex |

**Supplementary Table S3**

Histopathological scoring scheme.

| Score | Cornea | Conjunctiva | Iris/ciliary body | Vitreous | Retina |
| --- | --- | --- | --- | --- | --- |
| 0 | Normal | Normal | Normal | Normal | Normal |
| 1 | Focal thickening | Mild inflammation | Mild iritis | Occasional cells | Focal retinitis |
| 2 | Diffuse thickening | Moderate inflammation | Moderate iridocyclitis | Moderate cells or early fibrin membranes | Diffuse retinitis |
| 3 | Marked thickening | Marked inflammation | Severe iridocyclitis | Marked fibrinous membranes, cells | Marked necrosis |
